# Acute-Onset Quadriplegia Presenting With Hyperreflexia: A Dilemma in Diagnosis

**DOI:** 10.7759/cureus.60494

**Published:** 2024-05-17

**Authors:** Muskaan Ahlawat, Sachin Shivnitwar, Shubhangi Kanitkar, Sai Priya Ande, Akshata Borle

**Affiliations:** 1 Internal Medicine, Dr. D. Y. Patil Medical College, Hospital & Research Centre, Dr. D. Y. Patil Vidyapeeth (Deemed to be University), Pune, IND

**Keywords:** guillain-barré syndrome, autoimmune, deep tendon reflex, acute quadriplegia, intravenous immunoglobulins (ivig)

## Abstract

An autoimmune polyradiculoneuropathy, Guillain-Barré syndrome (GBS) is an acute, rapidly progressive, and fulminant one. Rapidly developing motor weakness along with absent reflexes, with or without sensory impairment, is the hallmark of GBS. GBS is never a hereditary entity; it is always acquired by the individual. Here, we present an interesting case of GBS in a 37-year-old male patient presenting with lower limb weakness for one day which had progressed to upper limb weakness in a day. There was a history of fever and loose stools four days back. On examination, vitals were within normal limits including single breath count. Central nervous system (CNS) examination revealed as follows: bicep jerk, tricep jerk, and supinator jerk were National Institute of Neurological Disorders and Stroke (NINDS) scale grade 2 in bilateral upper limbs. Knee jerk was NINDS scale grade 3 in bilateral lower limbs, which was unusual considering that GBS presents with areflexia or reduced reflexes. Ankle jerk was absent in bilateral lower limbs. Plantars were mute bilaterally. Nerve conduction study was suggestive of axonal and demyelinating motor neuropathy involving all four limbs. The patient was planned for intravenous immunoglobulin at a dose of 2 g/kg/day for five days but developed an allergic reaction to the first dose; hence, the therapy was discontinued, and the option of plasmapheresis was given to which the patient refused. This is a report of a case of GBS with hyperreflexia which is an uncommon entity in the Indian subcontinent.

## Introduction

Guillain-Barré syndrome (GBS) is an autoimmune polyradiculoneuropathy which is acute, often severe, and fulminant [1]. Acute inflammatory demyelinating polyneuropathy (AIDP) is the leading variant. Moreover, there are two subtypes, known as "axonal" or "nodal/paranodal" variations, called acute motor axonal neuropathy (AMAN) and acute motor sensory axonal neuropathy (AMSAN), which are often clinically severe. Although it is uncommon in the Indian subcontinent, there have been multiple cases of reflex conservation and hyperreflexia in individuals with the axonal variety of GBS in Chinese, Japanese, and European populations [2]. Deficits in cutaneous sensory perception, such as loss of pain and temperature perception, are often moderate; however, they more significantly impair functions supported by vast sensory fibers, such as proprioception and deep tendon reflexes. While severe cases of bladder dysfunction are possible, they are often temporary.

## Case presentation

A 37-year-old male patient, a farmer by occupation, presented with complaints of bilateral lower limb and upper limb weakness for one day which was acute on onset, progressive in nature, and started from lower limbs. The weakness progressed to bilateral upper limbs within one day and to such an extent that the patient was not able to walk or stand up from a sitting (squatting) position and carry out his daily activities. There was a history of fever and loose stools, multiple episodes, watery, non-bloody, two days prior to the onset of weakness. There was no history of trauma, fall, loss of sensations, abdomen pain, chest pain, shortness of breath, palpitations, cold, cough, etc. The patient had no comorbidities or any addictions. No significant drug history was present.

On examination, vital parameters including single breath count (50) were within normal limits.

On neurological examination, the patient was lucid, alert, and aware of the time, place, and person. There was no neck stiffness. Cranial nerve examination including facial nerve examination was within normal limits. The tone was within normal range in upper limbs with hypotonia in lower limbs. Power was Medical Research Council (MRC) grade 3 in bilateral upper limbs and MRC grade 2 in bilateral lower limbs. Hand grip was reduced bilaterally. Mental status examination was normal. Superficial reflexes were National Institute of Neurological Disorders and Stroke (NINDS) scale grade 2. Deep tendon reflexes, bicep jerk, tricep jerk, and supinator jerk, were NINDS scale grade 2 in bilateral upper limbs. Knee jerk was NINDS scale grade 3 in bilateral lower limbs. Ankle jerk was absent in bilateral lower limbs. Plantar reflex was mute bilaterally. Sensory system examination was within normal limits. Cerebellar signs were absent (Table 1).

**Table 1:** Summary of neurological examination

Examination	Right		Left	
	Upper limb	Lower limb	Upper limb	Lower limb
Tone	Normal	Hypotonia	Normal	Hypotonia
Power	Grade 3	Grade 2	Grade 3	Grade 2
Bicep jerk	Grade 2		Grade 2	
Tricep jerk	Grade 2		Grade 2	
Supinator jerk	Grade 2		Grade 2	
Knee jerk	Grade 3		Grade 3	
Ankle jerk	Absent		Absent	
Plantars	Mute		Mute	

Routine hematological and biochemical investigations revealed elevated total leucocyte count (TLC); all the rest were within normal limits including serum potassium levels. Creatinine phosphokinase levels were within normal limits (Table 2).

**Table 2:** Hematological investigations of the patient WBC: white blood cell; MCV: mean corpuscular volume; HIV: human immunodeficiency virus; HCV: hepatitis C virus; HBsAg: hepatitis B surface antigen; SGPT: serum glutamic pyruvic transaminase; SGOT: serum glutamic oxaloacetic transaminase; CPKNAC: creatine phosphokinase-N-acetyl cysteine

Parameter	Result
Hemoglobin	15.3 g/dL
WBC count	15,700/mcgL
Platelet	2,58,000/mcgL
MCV	85.9 fL
Sodium	140 mmol/L
Potassium	3.63 mmol/L
HIV/HCV/HBsAg	Negative
Bilirubin (total)	0.4 mg/dL
Bilirubin (direct)	0.17 mg/dL
SGPT	75 U/L
SGOT	45 U/L
Urea	27 mg/dL
Creatinine	0.7 mg/dL
CPKNAC	247

Nerve conduction studies were done which revealed axonal and demyelinating motor neuropathy involving all four limbs.

MRI whole spine screening was done to rule out the important differential diagnosis of a high cervical lesion, and it showed no significant abnormality. MRI brain was normal. A diagnosis of atypical GBS was considered.

The patient was planned for intravenous immunoglobulin (IVIG) therapy at a dose of 2 g/kg for five days. However, he developed an allergic reaction during the first dose of IVIG, and the therapy was discontinued. For the allergic reaction, the patient was given an injection of hydrocortisone 100 mg stat and Inj. Avil 1 ampoule stat and was observed for any deterioration. He was advised plasmapheresis (PLEX) following the reaction to IVIG for which he was not willing. Following this, the patient was given a multivitamin injection and supportive care. After two weeks, the patient's weakness improved, and he was able to walk and do his regular day-to-day activities. However, some residual weakness was still present. The patient was on regular follow-up, and he was able to go to his job and perform his daily activities. Residual weakness was still present due to which the patient had a limping gait.

## Discussion

The acute self-limited polyneuropathy known as GBS was initially outlined in 1916 by Guillain, Barré, and Strohl. It wasn't until the acute axonal variety of GBS was initially described in the 1980s that the illness was recognized as demyelinating. Since then, the two primary subgroups of GBS have been identified [3]. Although attenuated reflexes or absent reflexes are the hallmarks of GBS, preserved reflexes or hyperreflexia can rarely be found in GBS [4]. Forty-four sufficiently described cases with GBS and hyperreflexia were found in a systematic analysis of the literature from January 1, 1993, to August 30, 2019. Of these patients, 73.3% were from Japan, 6.7% were from the United States, and 6.7% were from India [5]. When GBS patients exhibit hyperreflexia, it is typically a sign of prior *Campylobacter jejuni* infection, with the majority of patients having experienced diarrhea and abdominal pain in the past [6]. In underdeveloped nations like India, access to antibody testing is restricted, which complicates diagnosis. AMAN, acute motor conduction block neuropathy, and acute facial diplegia with hyperreflexes are the types most frequently linked to retained or brisk reflexes [7]. Our patient had preserved and brisk reflexes throughout the duration of the illness. About three weeks following an acute viral event, usually respiratory or gastrointestinal, roughly 70% of cases of GBS arise. According to circumstantial evidence, all cases of GBS are thought to be the consequence of immune reactions to nonself antigens (vaccines, infectious organisms), which are misdirected to host nerve tissue via a process known as molecular mimicry. Gangliosides, a particular type of glycoconjugate, are most likely the targets in the brain. Among the first characteristics in nerve conduction investigation in AIDP are delayed F-wave latencies, extended distal latencies, and lower amplitudes of compound muscle action potentials (CMAPs). These most likely result from the early tendency for distal motor nerve terminals and nerve roots to become involved [1]. Subsequently, temporal dispersion, conduction block, and conduction velocity slowing may be observed.

The inhibition system in spinal interneurons is thought to be the mechanism in brisk deep tendon reflexes [4,8]. Rather than axonal degeneration, distal conduction abnormalities are thought to be the cause of the pathogenic process. This condition is known as acute motor conduction block neuropathy or reversible conduction failure [6,8]. Immune-mediated conduction failure at the Ranvier nodes without demyelination may cause conduction block [9].

Since both high-dose IVIG and PLEX are equally effective for treating typical GBS, these treatments can be started for GBS. Because of its high safety profile and convenience of administration, IVIG is frequently used as the first therapy. Combining PLEX and IVIG therapy has not been shown to be beneficial for patients whose first-line treatment failed [10]. IVIG was administered in five daily infusions for a total dose of 2 g/kg body weight [1]. Very mild cases of GBS may occasionally be treated conservatively without IVIG or PLEX, particularly in individuals who first seem to have achieved a plateau. Despite being in the acute phase of the disease and exhibiting considerable motor weakness, our patient made a full recovery without the need for IVIG or PLEX.

## Conclusions

A prevalent cause of acute peripheral neuropathy, GBS is characterized by hyporeflexia or areflexia. However, the preservation of reflexes or hyperreflexia should not rule out the diagnosis. A dilemma in diagnosis often delays treatment and recovery time for the patient, which is why it is important to spread awareness about the hyperreflexic variant of the disease. Since the time of therapy initiation is vital, diagnosis should be established as soon as possible and treatment started promptly. This case report is to increase awareness regarding the normo-/hyperreflexic variant of GBS.
